# Generation of plane spiral orbital angular momentum using circular double-slot Vivaldi antenna array

**DOI:** 10.1038/s41598-020-75202-6

**Published:** 2020-10-27

**Authors:** Yongzhong Zhu, Weiguo Dang, Xiaoyu Liu, Yijun Chen, Xiaofei Zhou, Hongyan Lu

**Affiliations:** College of Information Engineering, Engineering University of PAP, Xi’an, 710086 Shaanxi China

**Keywords:** Electrical and electronic engineering, Electronics, photonics and device physics

## Abstract

A novel and feasible solution to generate plane spiral orbital angular momentum (PSOAM) vortex beam is proposed in this paper. The general principle of generating PSOAM in circular antenna array (CAA) is deduced, and verified by the simulation of the dipole CAA. Four double-slot Vivaldi antenna elements connect sequentially and are inserted into a CAA, which makes the proposed antenna easily fabricated and fully miniaturized. Both simulated and measured results show that it can generate PSOAM with *l* = 3 (*l* is the mode number of vortex wave) at 5 GHz. The vortex phase of *l* = 3 is observed, the measured peak gain is 5.7 dB. The VSWR remains below 2 from 4 to 6 GHz, which shows the impedance bandwidth of S_11_ < −10 dB is more than 40%.

## Introduction

Orbital angular momentum (OAM) of different modes, as an inherent property of electromagnetic waves, can be theoretically multiplexed in an infinite orthogonal channel at a single frequency, which greatly alleviates the scarcity of radio spectrum resources^1,2^. Due to its helical phase front, electromagnetic wave with OAM has outstanding advantages in detecting and imaging, especially for the rotating target^3,4^. Consequently, it has aroused widespread interest, and design of practical OAM antenna has become a current focus. Because the 3D radiation pattern of current OAM antennas is a center-hollowed cone, and their E plane patterns are made up of two divergent beams, the two beams will separate further as the transmitting distance increases. In this case, the location and direction of the receiving antenna must be carefully configured in order to receive the OAM waves properly. Furthermore, for the OAM waves carrying different modes radiated by the same antenna, the divergent angle usually varies with the OAM mode number *l*^5,6^. Therefore, the receiving power of OAM signals carrying different modes will be different at the same location, which makes it even more difficult to multiplex different OAM modes in the wireless communication system.

**Figure 1:**
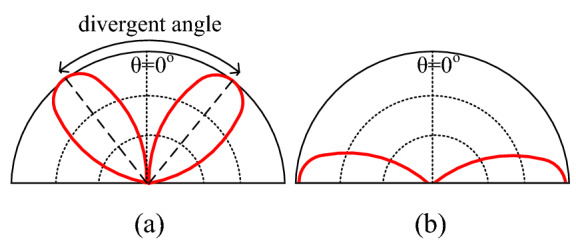
Comparison of E plane radiation pattern between (**a**) OAM and (**b**) PSOAM antenna.

Recently, the concept of PSOAM has been proposed^7,8^. The center hollow of its 3D radiation pattern can be enlarged by modifying the configuration of traditional OAM antenna. As shown in Fig. 1, the divergent angle in the E-plane pattern can be increased up to 180°, which indicates the omnidirectional side-fire pattern is obtained. Thus, it is far more convenient for receiving and de-multiplexing in practical applications. Because the PSOAM beam propagates along a two-dimensional plane helix, the phase singularity is located inside the antenna itself. Meanwhile, there is no energy black hole problem in the propagation path since the beam divergent angle remains same during the propagation. The PSOAM beam propagating along the transverse direction inherits the vorticity and orthogonality of the traditional OAM beam. The PSOAM beams with different modes have the same 180° divergence angle and can propagate in the same direction, which easily realize multi-mode synthesis, then avoid the problem of beam divergence inconsistency^9,10^. Although the PSOAM shows the above advantages over traditional OAM, current research on the PSOAM antenna is far from adequate.

So far, there are four generation methods of the PSOAM beam. Reference^11^ proposed a traveling-wave circular slot antenna^11^ with the characteristic of high modal purity. However, its large size makes it not conducive to multi-mode superposition. Reference^12^ proposed a ring dielectric resonator antenna loaded with meta-surface, which has disadvantages of large dimension, narrow beam, and narrow impedance bandwidth. In order to improve shortcomings of above antennas, a ring dielectric resonator antenna has been proposed in Ref.^13^, which has advantages of compact size and convenient beam superposition. However, to a certain extent, the low radiation efficiency of the antenna limits its development. A circular cylindrical conformal microstrip antenna array^13^ has been proposed in Ref.^14^, which has a compact structure and can generate multiple modes simultaneously, but the design of the feeding network is complex and bulky. In conclusion, the PSOAM antennas designed so far have shortages of narrow impedance bandwidth, low peak gain, and limited reconfigurability. In fact, the OAM antenna based on the uniform circular array (UCA) can realize various of characteristics using different array elements. Thus, considerable reconfigurability in performances like polarization and radiation pattern can be seen in it.

In this letter, PSOAM beam is realized by circular antenna array consists of wide beam directional element. The theory of generating PSOAM wave by CAA is deduced and verified. Then, a PSOAM Vivaldi CAA is designed and measured, which provides a new way for the design and realization of PSOAM antenna. Compared with the above conventional PSOAM antennas, the proposed antenna has better features, such as more compact structure, simpler feeding network, and wider impedance bandwidth.

### Theory of generating PSOAM by CAA

**Figure 2:**
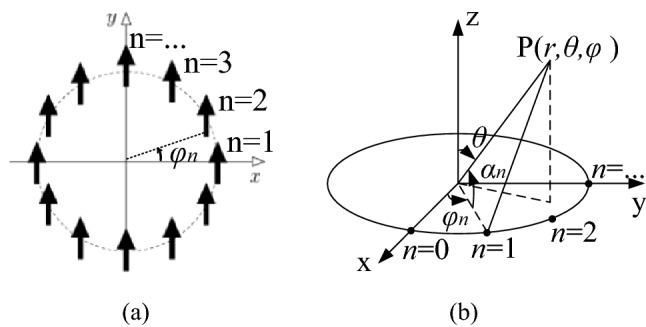
(**a**) Configuration of CAA. (**b**) Coordinate system for far field calculation.

The configuration of the CAA is shown in Fig. 2a. *a* is the radius of the CAA*.* The antenna elements are located on the XY plane with the azimuth angle of $\phi_{n} = 2\pi n/N$, where N is the number of array elements and n = 0, 1, 2, …, N − 1. The Feeding phase of each element is $\varphi_{n} = l\phi_{n}$, where *l* is the OAM mode number.

As shown in Fig. 2b, the center of CAA is taken as the phase reference point for calculating the wave path difference. The angle ${\upalpha }_{{\text{n}}}$ is formed by observing point P(r,${\uptheta }$,${\varphi }$) in the far field, the phase reference point, and the element n. Then, the wave path difference to P(r,$\uptheta$,${\varphi }$) between the phase reference point and the element n is $a\cos \alpha_{n}$. Thus, array factor, $F(\theta ,\phi )$, can be expressed as:

1 $$\begin{aligned} F(\theta ,\phi ) & = \sum\nolimits_{{{\text{n}} = 0}}^{N - 1} {e^{{j\varphi_{n} }} } e^{{jka\cos \alpha_{n} }} \\ & = \sum\nolimits_{n = 0}^{N - 1} {e^{{jl\phi_{n} }} e^{{jka\sin \theta \cos (\phi - \phi_{n} )}} } \\ \end{aligned}$$

**Figure 3:**
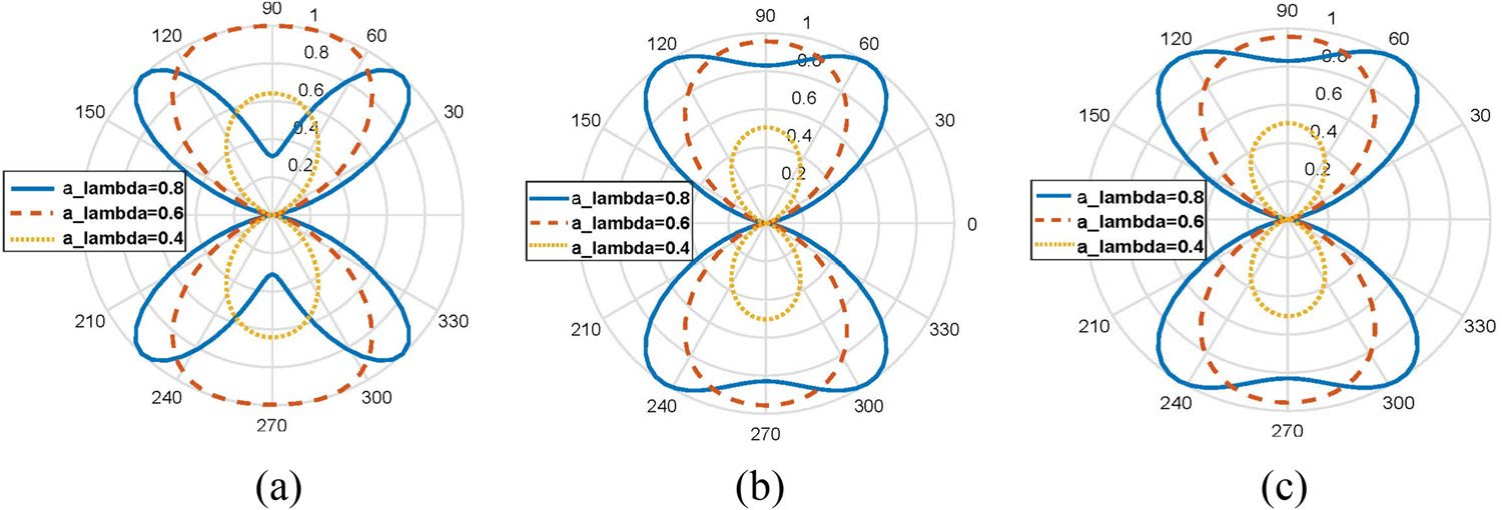
Comparison between the calculation results of $F\left( {\theta ,\varphi } \right)$ by Eq. (1) and Eq. (2) (**a**) Eq. (1), N = 8; (**b**) Eq. (1), N = 20; (**c**) Eq. (2), N = ∞.

When N is sufficiently large and $\Delta \phi = 2\pi /N$ is sufficiently small, the above equation can be dealt as an integral:

2 $$\begin{aligned} & \sum\nolimits_{n = 0}^{N - 1} {e^{{jl\phi_{n} }} } e^{{jka\sin \theta \cos (\phi - \phi_{n} )}} \\ & \quad = \frac{1}{\Delta \phi }\sum\nolimits_{n = 0}^{N - 1} {e^{{jl\phi_{n} }} } e^{{jka\sin \theta \cos (\phi - \phi_{n} )}} \Delta \phi \\ & \quad \approx \frac{N}{2\pi }\int_{0}^{2\pi } {e^{{jl\phi_{n} }} } e^{{jka\sin \theta \cos (\phi - \phi_{n} )}} d\phi_{n} \\ & \quad = Nj^{l} J_{l} (ka\sin \theta )e^{jl\phi } \\ & \quad = Nj^{l} J_{l} (2\pi a_{\lambda } \sin \theta )e^{jl\phi } \\ \end{aligned}$$

where $a_{\lambda } = a/\lambda$, $\lambda$ is the working wavelength of CAA in vacuum. By Eq. (2), the omnidirectional pattern of PSOAM can be realized when the required radius *a* is taken in a certain interval. Let $x_{l}$ be the first extreme point of the lth Bessel function: $J_{l}^{\prime } (x_{l} ) = 0$. According to Eq. (2), if $F(\theta ,\phi )$ gets maximum when $\theta = 90^{^\circ }$, there must be $2\pi a_{\lambda } = x_{l}$, and this array radius is denoted as $a_{\lambda 0} = x_{l} /2\pi$. When $a_{\lambda } > a_{\lambda 0}$, $J_{l} (2\pi a\sin \theta )$ will get its first extreme point at $0^{^\circ } < \theta < 90^{^\circ }$. That is to say, a traditional divergent pattern of the OAM antenna will be generated. And when $a_{\lambda }$ is very large, the first extreme point will be taken at ${\uptheta }$ very close to 0, which means that an OAM CAA with large array radius can concentrate the two divergent beams in the direction of $\theta = 0^\circ$. When $a_{\lambda } < a_{\lambda 0}$, will still get its first extreme point at $\theta = 90^\circ$. However, this extreme value is smaller than the value when $a_{\lambda } = a_{\lambda 0}$. The above analysis is validated in MATLAB R2015b, where $\left| {F(\theta ,\phi )} \right|$ with different $a_{\lambda }$ are plotted in E plane, as shown in Fig. 3. All results are plotted when *l* = 3, working frequency $f$ = 5 GHz, $\phi = 0^\circ$ and $a_{\lambda 0}$ = 0.669. It is also observed that the larger N is, the more similar the results from Eq. (1) and Eq. (2) will be.

**Figure 4:**
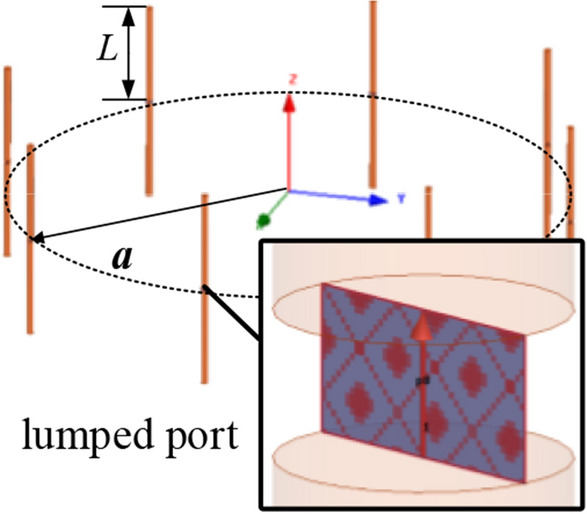
Simulated configuration of the PSOAM dipole CAA.

**Figure 5:**
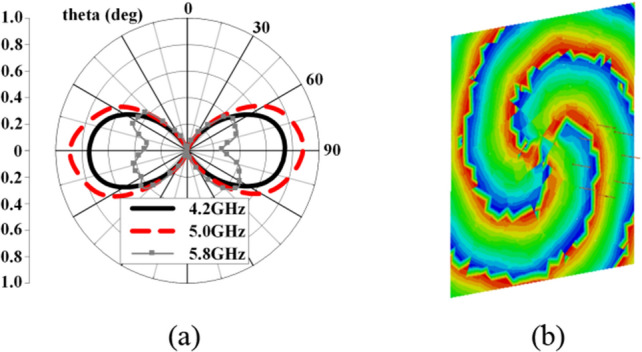
Simulated results of the PSOAM dipole CAA. (**a**) E plane field pattern. (**b**) Electric field phase vortex of *l* = 3.

In conclusion, when $a_{\lambda } < a_{\lambda 0}$, the array factor is the radiation pattern with characteristic of side-fire, PSOAM may be generated by CAA, where $a_{\lambda }$ is the electrical array radius, and $a_{\lambda 0}$ is determined by the desired PSOAM with mode number *l*. To verify the above theory, a dipole CAA at 5 GHz is simulated, as shown in Fig. 4. The length of the dipole arm is L = 13.04 mm, which is about $\lambda$/4. The array radius is a = $a_{\lambda 0} \cdot \lambda$ = 40.1 mm, where $a_{\lambda 0}$ = 0.669 (desired mode number *l* = 3). As shown in Fig. 5, the dipole CAA gets the optimal side-fire E-plane radiation pattern at $f_{0}$ = 5 GHz. When the working frequency is lower than $f_{0}$, the radiation pattern remains to be side-fire, while the gain decreases. When the working frequency is higher than $f_{0}$, the main beam direction begins to be elevated, and its radiation pattern becomes the divergent one of traditional OAM. These results are all in good agreement with the above analysis. Besides, an electric field vortex phase of *l* = 3 is observed on an observing plane 200 mm away from the array plane, which proves that PSOAM is successfully generated.

**Figure 6:**
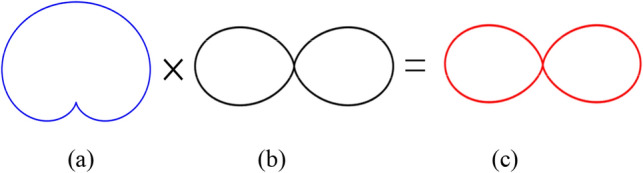
Vertical radiation patterns for the antenna (**a**) Wide beam antenna. (**b**) Array factor. (**c**) Circular antenna array.

The above studies prove that PSOAM is successfully generated by the dipole CAA, because the main beam direction of the dipole and the array factor of the UCA is the same to those of the PSOAM ($\theta = 90^{^\circ }$). Actually, the circular antenna array consists of directional elements can also generate PSOAM beam. As long as the directional radiation antenna element has a wide beam radiation pattern and the value is large enough at $\theta = 90^{^\circ }$, as shown in Fig. 6. If the half-power beamwidth (HPBW) of the antenna element could be widened, the gain for $\theta = 90^{^\circ }$ may be improved greatly. It can be seen from the result that the CAA has potential in generating PSOAM. In other words, selecting the array element reasonably can make the radiation pattern of the CAA obtain the maximum value in the side-fire direction and zero in the end-fire direction, which reflects the characteristics of the PSOAM pattern. Therefore, it can be inferred that the side-fire radiation pattern can be obtained by adjusting the CAA array element and its parameters, and verify the possibility that the CAA with end-fire antennas can generate PSOAM.

### Antenna design

**Figure 7:**
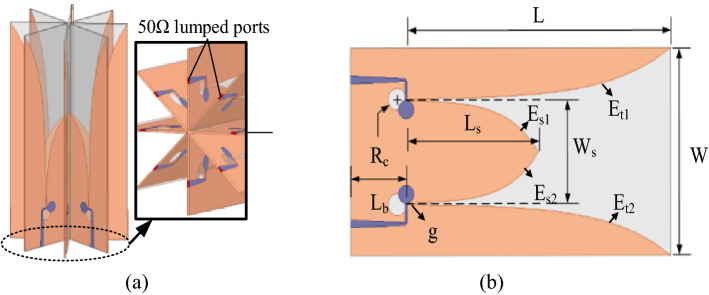
(**a**) Configuration of the simulating model of proposed Vivaldi CAA. (**b**) Parameters of Vivaldi antenna element.

To verify the above theory, a CAA consisting of four Vivaldi antenna elements with double-slot structure is designed. The antenna array is shown in Fig. 7a. This kind of element is similar to the differential feeding structure, which is beneficial to the cross welding of elements. As shown in Fig. 7b, a single double-slot Vivaldi antenna can be regarded as two elements because there are two feeding ports. Therefore, it is equivalent to the antenna array has eight elements. The curve equation with structural parameters to establish a double-slot Vivaldi antenna in Fig. 7b is as follows:

3 $${\text{E}}_{{{\text{s}}1}} :y = \frac{1}{2}\left( {W_{s} - g \times \exp \left( {\ln \left( {\frac{{W_{s} }}{g}} \right) \times \frac{t}{{L_{s} }}} \right)} \right)\quad (0 \le t \le L_{s} )$$

4 $${\text{E}}_{{{\text{s}}2}} :y = \frac{1}{2}\left( {g \times \exp \left( {\ln \left( {\frac{{W_{s} }}{g} \times \frac{t}{{L_{s} }}} \right) - W_{s} } \right)} \right)\quad (0 \le t \le L_{s} )$$

5 $${\text{E}}_{{{\text{t}}1}} :y = \frac{1}{2}\left( {W_{s} + g \times \exp \left( {\ln \left( {\frac{{W - W_{s} }}{g} \times \frac{t}{L}} \right)} \right)} \right)\quad (0 \le t \le L)$$

6 $${\text{E}}_{{{\text{t}}2}} :y = - \frac{1}{2}\left( {W_{s} + g \times \exp \left( {\ln \left( {\frac{{W - W_{s} }}{g}} \right) \times \frac{t}{L}} \right)} \right)\quad (0 \le t \le L)$$

The substrate of the proposed antenna is Teflon ($\varepsilon_{r}$ = 2.1) with the thickness h = 1 mm, other geometrical parameters are given in Table 1.

**Table 1:** Major parameters of Vivaldi antenna element.

Parameters	W	L	W_s_	L_s_	R_c_	L_b_	g
Values (mm)	54.4	81.6	27.2	40.8	3.1	17	0.34

**Figure 8:**
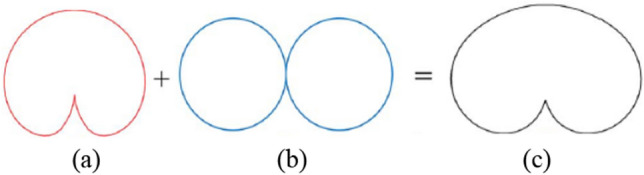
Vertical radiation patterns for the antennas (**a**) Vivaldi antenna. (**b**) Two adjacent elements. (**c**) Vivaldi antenna with two adjacent elements.

Since the Vivaldi antenna element has high directivity in an ultra-wideband^15,16^, it is difficult to radiate PSOAM beam. In order to solve this problem, the Vivaldi elements are embedded in a cross structure, where each element is greatly influenced by two adjacent elements. The two elements are equivalent to an omnidirectional monopole antenna, and its far-field radiation pattern obtains minimum and maximum gain when the elevation is $\theta = 0^{^\circ }$ and $\theta = 90^{^\circ }$ respectively. So if a Vivaldi antenna element is combined with the two adjacent elements, the gain in low elevation angle may be improved obviously, while that of $\theta = 0^{^\circ }$ angle is scarcely affected. Therefore, the vertical HPBW of the array element could be widened by the cross structure. Figure 8 demonstrates and verifies the influence on the radiation pattern by the cross structure.

**Figure 9:**
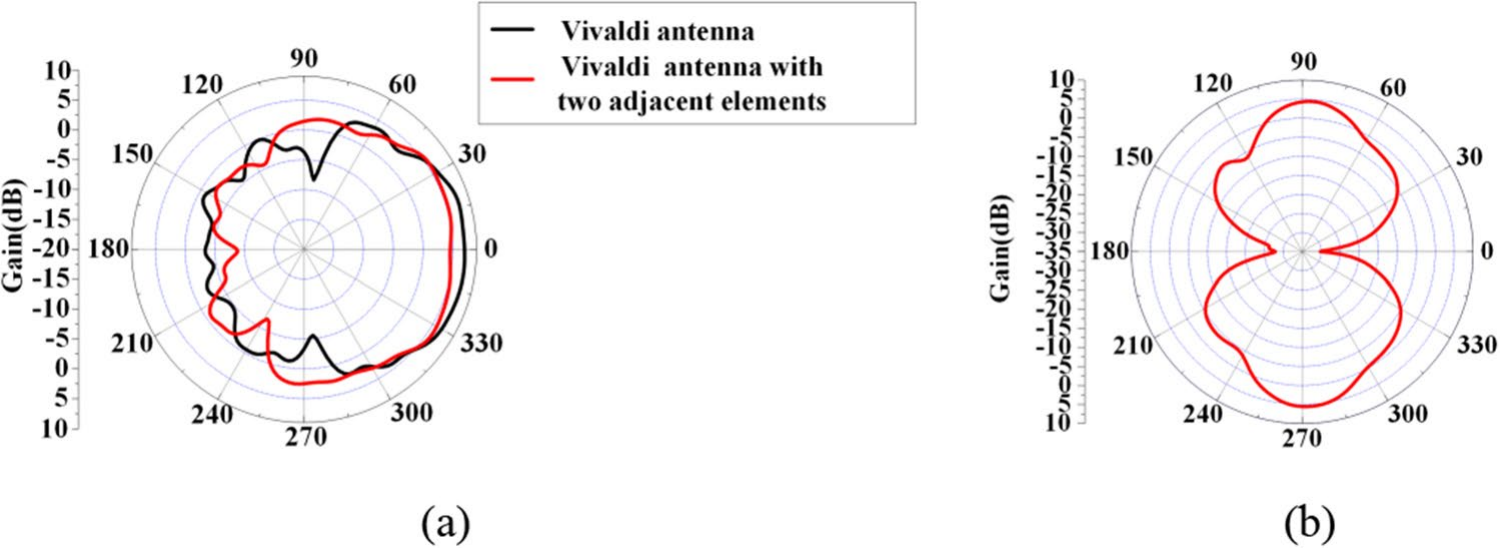
Simulated vertical radiation patterns for the antennas (**a**) Vivaldi antenna and Vivaldi antenna with two adjacent elements. (**b**) Vivaldi antenna array with eight elements.

According to the analysis in the above section, the 8-element Vivaldi PSOAM CAA with *l* = 3 at 5 GHz shown in Fig. 7 is designed. In the simulation by ANSYS HFSS 15, eight 50 ${\Omega }$-lumped ports are used to excite each antenna element independently. The phase difference between adjacent ports is $\Delta \phi = 2\pi l/N$ = 135°. The far-field radiation patterns of a single Vivaldi antenna and the Vivaldi antenna with two adjacent elements are shown in Fig. 9. It can be seen that the HPBW of the Vivaldi antenna element with two adjacent elements has been greatly expanded compared with a single Vivaldi element due to the mutual influence. In other words, the Vivaldi CAA has potential in generating PSOAM.

**Figure 10:**
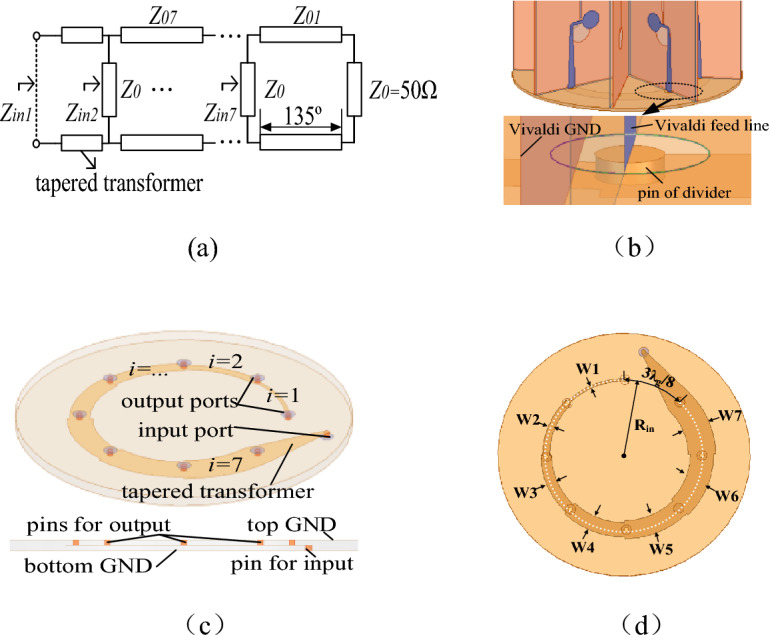
(**a**) Theoretical model. (**b**) Details of connection. (**c**) Structure configuration. (**d**) Major size parameters of the feeding network for Vivaldi CAA.

Then, a compact feeding network is designed for the above CAA. The feeding network is composed of 1 input port, 8 output ports, a tapered impedance transformer and 7 eighth-arc strip lines. The length of eighth-arc strip lines is $3\lambda_{g} /8$, which creates 135° phase difference. The eight output terminals of the feeding network are connected to eight Vivaldi elements. The schematic diagram of the sequential phase feeding network is depicted in Fig. 10a. $Z_{0i}$ (*i* = 1, 2……, 7) is the characteristic impedance of the *i*th segment with the width of W_i_ and length of $3\lambda_{g} /8$. Therefore, when the phase θ appears at output 2, phase differences $(\theta + 135^{^\circ } )$, ($\theta + 270^{^\circ }$), ……, ($\theta + 90^{^\circ }$) will be appearing at output 3 to output 8 respectively. Another principle that should be considered is the impedance matching in the design. Every strip line has the same electrical length of 135° at 5 GHz, providing a proper phase difference for *l* = 3. Since the input impedance $Z_{0}$ of each Vivaldi antenna is about 50 ${\Omega }$, the characteristic impedance of each strip line, $Z_{0i}$, is configured as $Z_{0i} = 50/i$ Ω. Thus, the input impedance of every port is 50Ω, namely $Z_{inm}$ = 50Ω, m = 1, 2, …7. Due to the input impedance of the Vivaldi antenna is about 50Ω, so the Z_L_ = 50Ω is substituted, and the characteristic impedance of each line can be solved, which are displayed in Table 2.

**Table 2:** Strip line characteristic impedance.

Characteristic impedance	Z_01_	Z_02_	Z_03_	Z_04_	Z_05_	Z_06_	Z_07_
Value (Ω)	50	25	16.7	12.5	10	8.3	7.1

According to the parameters in Table 2, the width of each segment of microstrip line can be calculated by Agilent commercial microwave calculation tool AppCAD, which are shown in Table 3.

**Table 3:** Major size parameters of the feeding network.

Parameters	W_1_	W_2_	W_3_	W_4_	W_5_	W_6_	W_7_	R_in_
Values (mm)	0.85	2.14	3.44	4.75	6.06	7.37	8.69	24.7

The theoretical circuit model of the feeding network is presented in Fig. 10a. The Fig. 10b demonstrates that the Vivaldi ground is connected with the upper metal ground of the feeding network, and it is fed by the feed line connected with the pin of divider through a ring hole on upper metal ground. As shown in Fig. 10c,d, the feeding network is composed of upper metal ground, a dielectric layer, a strip line layer, a dielectric layer and lower metal ground from top to bottom. The dielectric material is Teflon, whose thickness is 1 mm and permittivity $\varepsilon_{r}$ = 2.1, and dielectric loss tangent is about 0.001.

## Measurement and discussion

**Figure 11:**
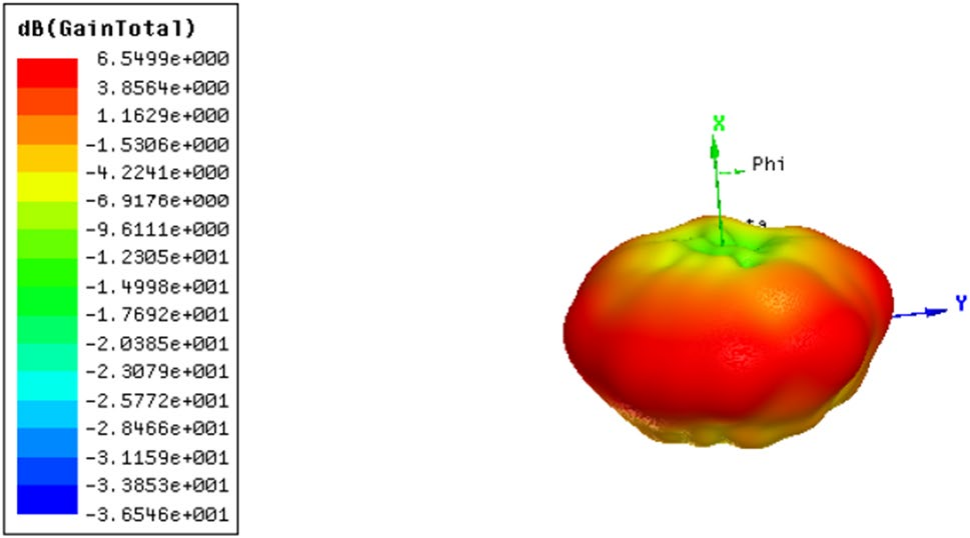
Simulated 3D radiation pattern of the proposed Vivaldi array.

**Figure 12:**
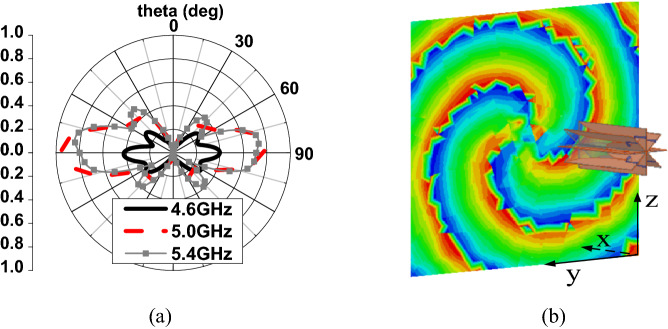
Simulated results of the PSOAM Vivaldi CAA. (**a**) E-plane radiation pattern. (**b**) Vortex phase of Ex.

The antenna is designed and simulated by Ansoft HFSS full-wave simulator based on the finite element method, according to the parameters listed in Table 1. The operating frequency is designed at 5 GHz. Figure 11 shows the simulated 3D radiation pattern of the Vivaldi antenna array. Figure 12 shows the E-plane radiation pattern and vortex phase of Ex, which proves that the vortex wave with *l* = 3 is generated successfully by the Vivaldi antenna array. The gain is also high (larger than 6 dB), which shows that the double-slot Vivaldi antenna as an array element has advantages in significantly improving the gain of the PSOAM antenna.

According to the analysis in Section II, a PSOAM-carrying radio beam with mode number *l* = 3 can be generated if the successive phase difference from element to element is stepped 0, 135°, 270°, 45°, 180°, 315°, 90°, 225°. A prototype Vivaldi CAA is then fabricated and measured. As shown in Fig. 9b, simulated results of the PSOAM Vivaldi CAA are agreement with the above analysis. The PSOAM radiation pattern is achieved, and the vortex phase with *l* = 3 is observed at 5 GHz. The measured VSWR < 2 is more than 40% (4 to 6 GHz). Compared with the PSOAM antenna consists of side-fire elements, for instance, the dipoles mentioned in the above analysis, the proposed PSOAM antenna has a large mutual influence, and its E-plane radiation pattern has some distortions.

**Figure 13:**
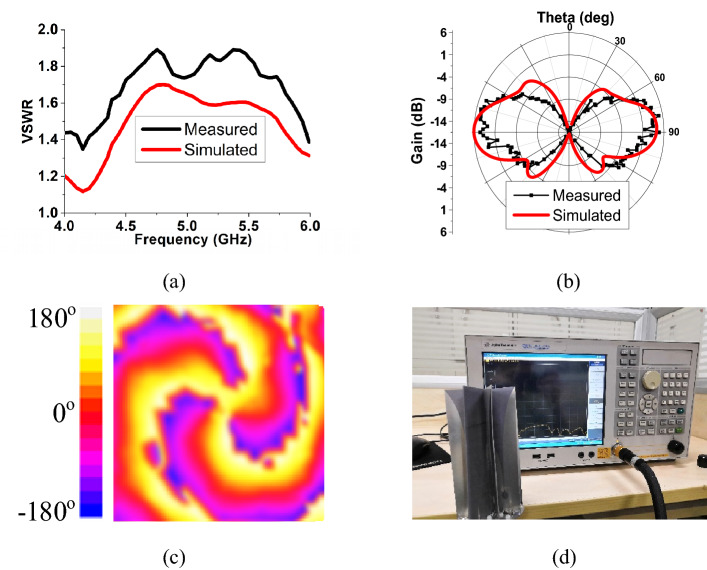
(**a**) Simulated and measured VSWR of Vivaldi CAA. (**b**) Simulated and measured E- plane radiation pattern at 5 GHz. (**c**) Measured vortex phase of Ex at 5 GHz. (**d**) The fabricated prototype and VNA Agilent E5071C used in measurement.

In Fig. 13a, the measured VSWR is in agreement with and less than 0.3 higher than the simulated one, which is within the reasonable range of fabricating and measuring errors. Figure 13b shows that both the simulated and the measured radiation patterns in the E-plane are side-fire. While the simulated peak gain is 6 dB, and the measured is about 5.7 dB. Figure 13c shows the vortex phase of Ex generated by the prototype antenna at 5 GHz. The vortex phase is measured by a near-field plane scanning technique. The fabricated prototype and VNA Agilent E5071C used in measurement are shown in Fig. 13d. The observation plane is 0.3 m $\times { }$ 0.3 m, 0.5 m away from the antenna’s top. The phase of E-field component vertical to the scanning plane, Ex, is measured by a standard measuring probe. Although some discontinuities occur due to noise and measuring error, 3 vortex arms are still clearly observed, which proves the generation of PSOAM.

After comparing the reported PSOAM antennas in terms of the center frequency, VSWR, total size, peak gain and feeding method in Table 4, it is easy to conclude that the currently existing PSOAM antennas are limited by impedance bandwidth, total size or peak gain, which are the key factors restricting applications in wireless communication system. Differently, the impedance bandwidth (VSWR < 2) of the proposed PSOAM antenna is more than 40% and the measured peak gain is about 5.7 dB, which are distinct advantages among the reported PSOAM antennas. Furthermore, the Vivaldi PSOAM antenna, with a compact structure, realizes PSOAM wave based on a novel UCA, which provides an unusual way to generate PSOAM and lays the foundation for future research on reconfigurability.

**Table 4:** Comparison with the reported PSOAM antennas.

*References	*f* (GHz)	VSWR < 2	Total size	Peak gain (dB)	Feeding method
[14]	10.1	2.8%	**S**_**a**_: 0.67 $\lambda_{0}$* 0.67 $\lambda_{0}$*0.95 $\lambda_{0}$ 222**S**_**f**_ : 8.75 $\lambda_{0}$* 5.72 $\lambda_{0}$	–	8 × 8 Butler Feed network
[11]	10	0.3%	**S**_**a**_: 4.12 $\lambda_{0}$* 4.12 $\lambda_{0}$*1.66 $\lambda_{0}$	3.71	90° hybrid coupler
[12]	15	0.2%	**S**_**a**_: 6.38 $\lambda_{0}$* 6.38 $\lambda_{0}$*0.5 $\lambda_{0}$	5.74	Coaxial line
[13]	10	0.3%	**S**_**a**_:0.90 $\lambda_{0}$* 0.90 $\lambda_{0}$*0.23 $\lambda_{0}$	3.013	Coaxial line
This paper	5	> 40%	**S**_**a**_: 0.90 $\lambda_{0}$* 0.90 $\lambda_{0}$* 1.64 $\lambda_{0}$ **S**_**f**_ : 0.90 $\lambda_{0}$* 0.90 $\lambda_{0}$* 0.016 $\lambda_{0}$	5.7	Sequential rotation feeding network

*S_**a**_**:** Size of the antenna. *S_**f**_** :** Size of the feeding work.

## Conclusion

In this letter, the theory and design principle of the PSOAM antenna based on CAA are deduced. Then, a compact double-slot Vivaldi CAA is proposed, which can generate PSOAM with mode number *l* = 3 at 5 GHz, with the VSWR < 2 more than 40% and the measured peak gain of 5.7 dB, benefiting the practical applications of OAM in fields like wireless communication and radar detecting.
